# Antarctic Krill Lipid and Fatty acid Content Variability is Associated to Satellite Derived Chlorophyll *a* and Sea Surface Temperatures

**DOI:** 10.1038/s41598-020-62800-7

**Published:** 2020-04-08

**Authors:** Nicole Hellessey, Robert Johnson, Jessica A. Ericson, Peter D. Nichols, So Kawaguchi, Stephen Nicol, Nils Hoem, Patti Virtue

**Affiliations:** 1 Institute for Marine and Antarctic Studies, University of Tasmania, 20 Castray Esplanade, Battery Point, Tasmania 7004 Australia; 2 CSIRO Oceans and Atmosphere, Castray Esplanade, Battery Point, Tasmania 7004 Australia; 3 Antarctic Climate and Ecosystems Cooperative Research Centre, 20 Castray Esplanade, Battery Point, Tasmania 7004 Australia; 4 School of Biological Sciences, Georgia Institute of Technology, 311 Ferst Drive NW, Atlanta, Georgia 30332 United States of America; 5 Cawthron Institute, Private Bag 2, Nelson, 7041 New Zealand; 6 Australian Antarctic Division, 203 Channel Highway, Kingston, Tasmania 7050 Australia; 7 Aker BioMarine Antarctic AS, Oksenøyveien 10, P.O. Box 496, NO-1327 Lysaker, Norway

**Keywords:** Lipidomics, Environmental monitoring, Marine biology, Marine chemistry

## Abstract

Antarctic krill (*Euphausia superba*) are a key component of the Antarctic food web with considerable lipid reserves that are vital for their health and higher predator survival. Krill lipids are primarily derived from their diet of plankton, in particular diatoms and flagellates. Few attempts have been made to link the spatial and temporal variations in krill lipids to those in their food supply. Remotely-sensed environmental parameters provide large-scale information on the potential availability of krill food, although relating this to physiological and biochemical differences has only been performed on small scales and with limited samples. Our study utilised remotely-sensed data (chlorophyll *a* and sea surface temperature) coupled with krill lipid data obtained from 3 years of fishery-derived samples. We examined within and between year variation of trends in both the environment and krill biochemistry data. Chlorophyll *a* levels were positively related to krill lipid levels, particularly triacylglycerol. Plankton fatty acid biomarkers analysed in krill (such as n-3 polyunsaturated fatty acids) increased with decreasing sea surface temperature and increasing chlorophyll *a* levels. Our study demonstrates the utility of combining remote-sensing and biochemical data in examining biological and physiological relationships between Antarctic krill and the Southern Ocean environment.

## Introduction

Antarctic krill (*Euphausia superba*, hereon krill) are at the centre of the wasp-waisted Southern Ocean ecosystem^1,2^. Krill, due to their high lipid (oil) content (up to 40% dry weight^3,4^), are vital food for predators in the region^5,6^. Krill have a naturally varied diet ranging from copepods and phytoplankton such as diatoms and flagellates, to marine snow and even cannibalism in harsh winter conditions^4,7–9^. Krill are predominantly herbivorous during the summer and are more omnivorous from autumn to spring^7^. Krill diet has been assessed through several different means such as microscopy^10^, DNA extraction^11,12^ and the use of signature fatty acid biomarkers^7,9^.

Biomarkers, such as fatty acids, have been used to examine krill health and diet previously^7,9,13–18^, as they are a reliable way of looking at the long-term diet of krill^10–12^. Fatty acid biomarkers are useful as they not only broadly classify what krill are eating, but their relative and absolute amounts allow insights into how much of these prey items and types krill have consumed over a more extended period of time^7,19,20^. Omega-3 (n-3) long-chain (≥C_20_) polyunsaturated fatty acids (n-3 LC-PUFA) are mainly derived from phytoplankton^21,22^, and are needed for krill health, growth and reproduction. They also serve as useful biomarkers^7,23,24^ in food-chain research. In particular, eicosapentaenoic acid (EPA; 20:5n-3) and docosahexaenoic acid (DHA; 22:6n-3), which are known to be associated with the intake of diatoms and dinoflagellates respectively^7,13^, are needed for production of krill eggs before spawning, and for the development of the larval krill^25^. Other sources for EPA and DHA may also exist. The specific source(s) of other n-3 LC-PUFA such as eicosatetraenoic acid (ETA, 20:4n-3) and docosapentaenoic acid (DPA, 22:5n-3) are not as well defined. EPA and DHA are consistently abundant in krill and make up a large part of the krill fatty acid profile^7,26^, particularly in summer and autumn. EPA and DHA are also the n-3 LC-PUFA targeted by the krill fishing industry for application into nutraceutical products^27–29^. The n-3 shorter chain PUFA, stearidonic acid (SDA, 18:4n-3), which is a flagellate marker, also plays a vital role in krill diet, although its precise function is not well understood^7^.

Phytoplankton, such as diatoms and dinoflagellates, all naturally produce chlorophyll which can be remotely detected via satellites^30–32^ using ocean colour data. Recent studies have shown that the colour of the ocean, due to shifts in the assemblage of these phytoplankton blooms^33^, is changing with climate change^34^. As these phytoplankton assemblages change, krill diet may also be altered as the climate changes^33,35^. Phytoplankton biomarker levels will have more pronounced changes within krill diet as lower trophic level populations will shift more rapidly with climate change^33^ than higher trophic levels^36^, such as grazers like copepods and krill^37^. Therefore, the biochemical composition of krill may shift year round from a winter diet which includes copepods^7^ (omnivorous) to a summer diet (herbivorous) for a larger part of the year; this shift occurs as sea ice is lost and waters warm (increase in sea surface temperature, SST) and become more acidic, allowing for greater phytoplankton blooms to occur^33,38,39^ year round. It is difficult, however, to link changes in the marine environment to changes in krill biochemistry *in situ*. Controlled aquarium experiments are difficult to conduct over longer time scales and cannot emulate conditions over large geographic areas. Understanding these large-scale relationships requires data collected over wide areas and long timeframes. Hence, using remotely-sensed satellite chlorophyll *a* (Chl *a*) data as a proxy for primary production is optimal for data collection that can happen simultaneously over large geographic areas and span over long timeframes^32,38^. Similarly, changes in phytoplankton blooms during spring and summer are influenced by vertical mixing and sea ice melt^40–43^ and SST in the Southern Ocean^44,45^; increases in SST are a major cause of ecosystem level shifts (e.g. phytoplankton assemblage change) with climate change^33,38,39^. Remotely-sensed SST data from satellites can also be collected over large geographic areas and long timeframes. Unfortunately, as of yet, a proxy for zooplankton, marine snow or bacterial assemblages that can be detected via satellite are not available. However, biological and biochemical data from krill samples are able to be collected and analysed over a large region of the Southern Ocean and over a long timeframe by the krill fishery, such as in Tarling, *et al*.^46^.

The krill fishery has been operating since the early 1970’s^47^, and in the mid-1990s the industry began to produce and sell krill oil as a nutraceutical due to its high levels of omega-3 containing oils^27^. The krill fishery operates year-round in the South Atlantic Ocean (Commission for the Conservation of Antarctic Marine Living Resources (CCAMLR) Area 48) and in particular near the West Antarctic Peninsula (WAP, CCAMLR Sub-Area 48.1), South Orkney Islands (SOI, CCAMLR Sub-Area 48.2) and South Georgia (SG, CCAMLR Sub-Area 48.3). Over the last few years the total catch has been approximately 300,000 tonnes of krill a year, and from a scientific research perspective the commercial harvest is a useful source of biological samples^7,46,48^. Satellites collect environmental data such as SST, ocean colour, sea surface height, fluorescence, wind direction and wind speed. While satellite data must be calibrated and validated, it can fill in gaps of data collected *in situ* and provides wide-area coverage. Linking remotely sensed environmental data with analysis of krill samples collected by the krill fishery is a cost-effective approach to explore biological responses to environmental changes.

Our study uses three consecutive years of krill lipid data from fishery-derived samples collected throughout the South Atlantic Ocean. We simultaneously accessed satellite data to link krill signature biochemical data to environmental conditions seen at the location of krill collection. We hypothesise that changes in krill biochemistry, specifically their fatty acid dietary biomarkers, will track broad scale satellite-derived environmental data. Linking environmental drivers to krill diet will assist in ecosystem energy budget and food web models. By basing such models around environmental parameters, future environmental scenarios can be modelled, and krill diet and ecosystem responses will be more reliably predicted.

We aim to show if a link between the environment and krill diet exists by examining whether: (1) SST (°C) and chlorophyll *a* levels (Chl *a*, mg m^−2^) are correlated to total lipid (mg g^−1^ krill dry weight) or phytoplankton fatty acid biomarkers (percentage (%) and mass (ug)) in a meaningful manner, (2) Chl *a*, total lipid and phytoplankton fatty acid biomarkers increase as SST decreases throughout summer and autumn, post the annual spring phytoplankton blooms and (3) larger shifts in SST and Chl *a* closer to the pole (currently an area in a state of flux) will drive larger shifts in krill biochemistry.

## Results

### Sea surface temperatures

During this study (Jan 2014 – Sep 2016) satellite-derived sea surface temperatures had the greatest variability in the South Orkney Islands (SOI), ranging from −1.28 to 1.93 °C (average 0.33 ± 0.86 °C), followed by South Georgia (SG) where temperatures ranged from −1.10 to 1.49 °C (average 0.49 ± 0.54 °C) and the West Antarctic Peninsula (WAP) which ranged from −1.28 to 0.94 °C (average −0.25 ± 0.78 °C). Temperatures decreased from summer into autumn in the WAP and SOI, and from the start of winter to spring in SG. However, in 2016, SST at WAP increased from summer into autumn and then decreased from early autumn onwards (Supplementary Fig. 1).

### Chlorophyll *a* concentrations

The levels of Chl *a* varied depending on the location and the season (Supplementary Fig. 2). Values of more than 5.78 mg m^−2^ of Chl *a* were observed in the SOI, and as low as 0.19 mg m^−2^ Chl *a* in SG. Chl *a* increased in summer and decreased rapidly during the autumn of 2014 around the SOI. Chl *a* concentrations in other years were more consistent, although very few satellite measurements were recorded at SG due to the time of year, cloud cover, ice cover and suboptimal sun angle.

Chl *a* varied the most around the SOI, ranging from 0.24 to 5.78 mg m^−2^ (average 0.88 ± 0.85 mg m^−2^), followed by the WAP which ranged from 0.20 to 1.34 mg m^−2^ (average 0.62 ± 0.30 mg m^−2^), and SG which ranged from 0.19 to 0.62 mg m^−2^ (average 0.39 ± 0.11 mg m^−2^). SG had the smallest range of Chl *a* concentrations and the lowest average Chl *a* concentration. SOI had the biggest range of Chl *a* concentrations and the highest average Chl *a* concentration.

### Geographic distribution

**Figure 1:**
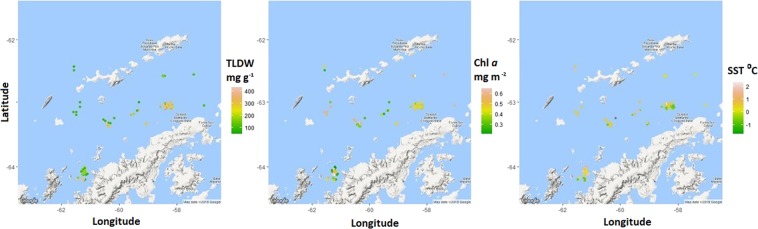
The geographic distribution of krill total lipid (mg g^−1^ dry weight, TLDW), the chlorophyll *a* (Chl *a*) concentration (mg m^−2^) and the sea surface temperature (°C, SST) of *Euphausia superba* samples collected in the West Antarctic Peninsula. Locations are points that krill were harvested by *FV Saga Seas* from January to May 2014–2016. Maps were produced using the RStudio (version 1.0.153^©^ 2017) package ggmaps (Kahle and Wickham, http://journal.r-project.org/archive/2013-1/kahle-wickham.pdf). Map data^©^ 2018 Google.

**Figure 2:**
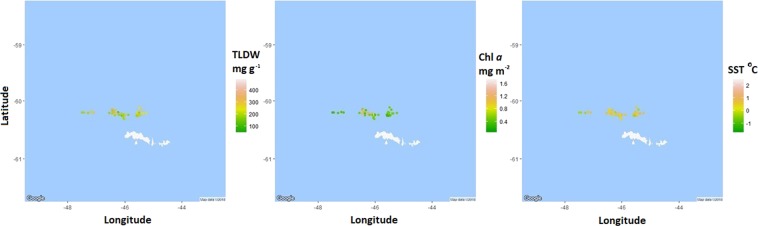
The geographic distribution of krill total lipid (mg g^−1^ dry weight, TLDW), the chlorophyll *a* (Chl *a*) concentration (mg m^−2^) and the sea surface temperature (°C, SST) of *Euphausia superba* samples collected in the South Orkney Islands. Locations are points that krill were harvested by *FV Saga Seas* from January to May 2014–2016. Maps were produced using the RStudio (version 1.0.153^©^ 2017) package ggmaps (Kahle and Wickham, http://journal.r-project.org/archive/2013-1/kahle-wickham.pdf). Map data^©^ 2018 Google.

**Figure 3:**
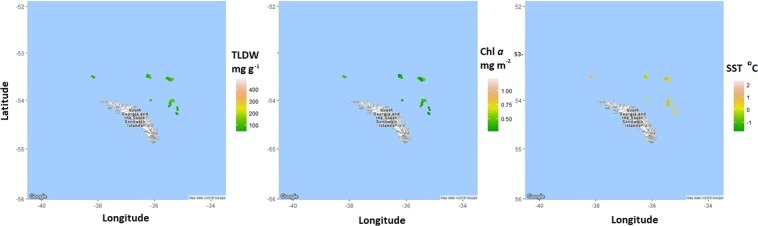
The geographic distribution of krill total lipid (mg g^−1^ dry weight, TLDW), the chlorophyll *a* (Chl *a*) concentration (mg m^−2^) and the sea surface temperature (°C, SST) of *Euphausia superba* samples collected at South Georgia. Locations are points that krill were harvested by *FV Saga Seas* from June to September 2014–2016. Maps were produced using the RStudio (version 1.0.153^©^ 2017) package ggmaps (Kahle and Wickham, http://journal.r-project.org/archive/2013-1/kahle-wickham.pdf). Map data^©^ 2018 Google.

The geographic distribution of SST, Chl *a* and krill total lipid (mg g^−1^ dry weight, TLDW), for CCAMLR Sub-Areas 48.1, 48.2 and 48.3 can be seen in Figs. 1, 2 and 3. Higher SST and Chl *a* in the Bransfield Strait (WAP) reflect bathymetric and current features (Fig. 1) as well as the northern advection of water from the deep canyon to the south of the SOI^49^ (Fig. 2). These oceanographic features are known locations of higher SST and Chl *a* that occur within the SG and SOI areas. Krill TLDW is also higher in the Bransfield Strait (Fig. 1). To the northwest of SG, higher SST and lower Chl *a* values can be seen due to the faster flowing current originating off the slope of SG (Fig. 3).

### Lipid and fatty acid general trends

Results describing the trends seen in krill TLDW, lipid classes and their fatty acids can be found in Ericson, *et al*.^7^ and Hellessey, *et al*.^48^. Briefly, krill TLDW and triacylglycerol (TAG) percentage (%) increased throughout summer to reach autumn highs in the WAP and SOI, whereas krill at SG had declining TLDW and TAG % throughout winter and spring. EPA and DHA (mg g^−1^ dry weight) followed the same seasonal trend as TLDW and TAG %. 16:1n-7c and SDA had variable quantities across all seasons, years and fishing locations. In summer, krill had high levels (% total fatty acids) of EPA, DHA and PUFA, but low 18:1n-9c/18:1n-7c ratios, indicating a more herbivorous diet.

### Tracking fatty acid biomarkers using satellite derived environmental data

Clear seasonal trends can be seen in fatty acid biomarkers in krill throughout the fishing seasons, both in percentage composition and quantitative amounts (mass, ug). Most diatom-based markers in krill (such as EPA, 16:4n-1 and 16:1n-7c) increased in percentage throughout summer to reach autumn highs in the WAP and SOI, as did 16:0 percentages. In comparison, krill at SG had declining diatom-based markers and 16:0 percentages throughout winter and spring. Dinoflagellate markers in krill such as DHA and SDA showed similar trends to diatom markers in their percentages. Both diatom and flagellate markers in krill showed the opposite trend in their masses (low in summer and autumn, higher in winter/spring), but this could also be due to location of sampling (WAP/SOI in summer/autumn and SG in winter/spring). Table 1 provides the P-value for 1-way ANOVAs comparing models as well as the adjusted r^2^ value and χ^2^ value for the associated model of best fit between the krill biochemical data (lipid and fatty acid content (mass) and composition (percentage)) and the environmental data (SST and Chl *a)* and their interaction terms from the South Atlantic region. Supplementary Tables 1–3 show these same relationships broken down into the smaller CCAMLR management sub-areas (SG, SOI and WAP, respectively).

**Table 1:** *Euphausia superba* total lipid (mg g^−1^ dry weight, TLDW), and lipid class composition (phospholipid (PL) and triacylglycerol (TAG) percentage) and fatty acid (20:5n-3 (EPA), 22:6n-3 (DHA), and 18:4n-3 (SDA)) percentage composition (%) and mass (ug) per krill against sea surface temperature (SST), chlorophyll *a* (Chl *a*) and their interaction terms for all seasons and pooled locations across the South Atlantic sector.

	SST	Chl *a* (overall)	Chl *a* (8D 3 × 3)	SST*Chl *a* (overall)	SST*Chl *a* (8D 3 × 3)
TLDW (mg g^−1^)	<0.001 (*0.144*) **0.086**	0.948 (−*0.004*) **0.018**	0.434 (−*0.005*) **0.238**	0.808 (*0.045*) **0.082**	0.007 (*0.134*) **0.238**
PL %	0.296 (*0.000*) **0.003**	<0.0001 (*0.061*) **0.006**	<0.0001 (*0.255*) **0.250**	0.176 (*0.069*) **0.029**	0.074 (*0.376*) **0.249**
TAG %	0.001 (*0.026*) **0.001**	0.344 (−*0.000*) **<0.001**	0.035 (*0.043*) **0.087**	0.480 (−*0.006*) **0.007**	0.015 (*0.193*) **0.087**
EPA %	0.001 (*0.025*) **0.080**	0.426 (−*0.002*) **0.018**	0.006 (*0.078*) **0.241**	<0.0001 (*0.085*) **0.241**	0.372 (*0.068*) **0.241**
EPA (ug)	<0.001 (*0.167*) **0.239**	0.664 (−*0.004*) **0.239**	0.667 (−*0.010*) **0.238**	0.385 (*0.051*) **0.239**	0.058 (*0.074*) **0.238**
DHA %	<0.001 (*0.065*) **0.082**	0.109 (*0.007*) **0.081**	0.194 (*0.009*) **0.240**	0.203 (*0.059*) **0.242**	0.918 (−*0.016*) **0.240**
DHA (ug)	<0.001 (*0.158*) **0.239**	0.534 (−*0.003*) **0.239**	0.102 (*0.021*) **0.238**	0.851 (*0.067*) **0.239**	0.048 (*0.117*) **0.238**
SDA %	0.078 (*0.006*) **0.003**	0.626 (−*0.003*) **0.003**	0.446 (−*0.005*) **0.240**	0.013 (*0.024*) **0.082**	0.612 (*0.189*) **0.240**
SDA (ug)	<0.001 (*0.141*) **0.239**	0.603 (−*0.003*) **0.239**	0.921 (−*0.012*) **0.238**	0.397 (*0.052*) **0.239**	0.422 (*0.096*) **0.238**
16:0%	0.001 (*0.037*) **0.083**	0.588 (−*0.003*) **0.082**	0.943 (−*0.012*) **0.244**	0.034 (*0.034*) **0.243**	0.417 (−*0.018*) **0.244**
16:0 (ug)	<0.001 (*0.182*) **0.239**	0.920 (−*0.004*) **0.239**	0.555 (−*0.008*) **0.238**	0.759 (*0.068*) **0.239**	0.091 (*0.042*) **0.239**
16:4n-1%	0.925 (−*0.003*) **<0.001**	0.451 (−*0.002*) **<0.001**	0.334 (−*0.000*) **0.019**	0.939 (-*0.010*) **0.003**	0.174 (*0.126*) **0.019**
16:4n-1 (ug)	<0.001 (*0.103*) **0.239**	0.407 (−*0.001*) **0.239**	0.337 (−*0.000*) **0.238**	0.787 (*0.008*) **0.239**	0.899 (*0.125*) **0.238**
16:1n-7c %	0.115 (*0.004*) **0.082**	0.982 (−*0.004*) **0.080**	0.583 (−*0.009*) **0.241**	0.061 (*0.015*) **0.241**	0.989 (−*0.034*) **0.241**
16:1n-7c (ug)	<0.001 (*0.143*) **0.239**	0.972 (−*0.004*) **0.239**	0.456 (−*0.005*) **0.238**	0.539 (*0.059*) **0.239**	0.069 (*0.043*) **0.238**
16:1/16:0 ratio (ug)	0.588 (−*0.002*) **0.239**	0.781 (−*0.004*) **0.239**	0.543 (−*0.008*) **0.238**	0.136 (*0.006*) **0.239**	0.950 (−*0.034*) **0.238**
EPA/DHA ratio (ug)	0.621 (*−0.002*) **0.239**	0.944 (−*0.004*) **0.239**	0.008 (*0.073*) **0.238**	0.730 (−*0.012*) **0.239**	0.625 (*0.060*) **0.238**
Phytanic acid %	0.013 (*0.014*) **<0.001**	0.047 (*0.013*) **<0.001**	0.149 (*0.014*) **<0.001**	0.309 (*0.012*) **<0.001**	0.362 (*0.098*) **<0.001**
Phytanic acid (ug)	0.009 (*0.016*) **<0.001**	0.052 (*0.012*) **<0.001**	0.149 (*0.014*) **<0.001**	0.313 (*0.009*) **<0.001**	0.250 (*0.127*) **<0.001**

Chl *a* was measured at both an overall scale (overall) and an 8-day 3 km × 3 km (8D 3 × 3) pixel scale for the entire South Atlantic sector. Values given are for: P values, r^2^ values (italics) and χ^2^ values (bold) for the model of best fit.

### Using environmental parameters as predictors of fatty acid biomarkers

The WAP had the most models that fit environmental factors with a significant relationship to the biomarkers in the krill found in that region (Supp. Table 3). The SG region had the next highest number of models that fit the biomarker/environment interaction relationship (Supp. Table 1). Both the overall South Atlantic area and the SOI had models that had less significant effects and correlations between the krill dietary biomarkers and the environment in those regions (Table 1 and Supp. Table 2). Based on these simple models, the best areas (due to their consistency and predictability in environmental factors) for use to examine krill dietary biomarkers are the WAP and SG, and the best environmental predictors for krill diet are SST, Chl *a* (8D 3 × 3) concentrations and the interaction of SST and Chl *a* (CCAMLR) concentrations (see Supplementary Materials).

**Figure 4:**
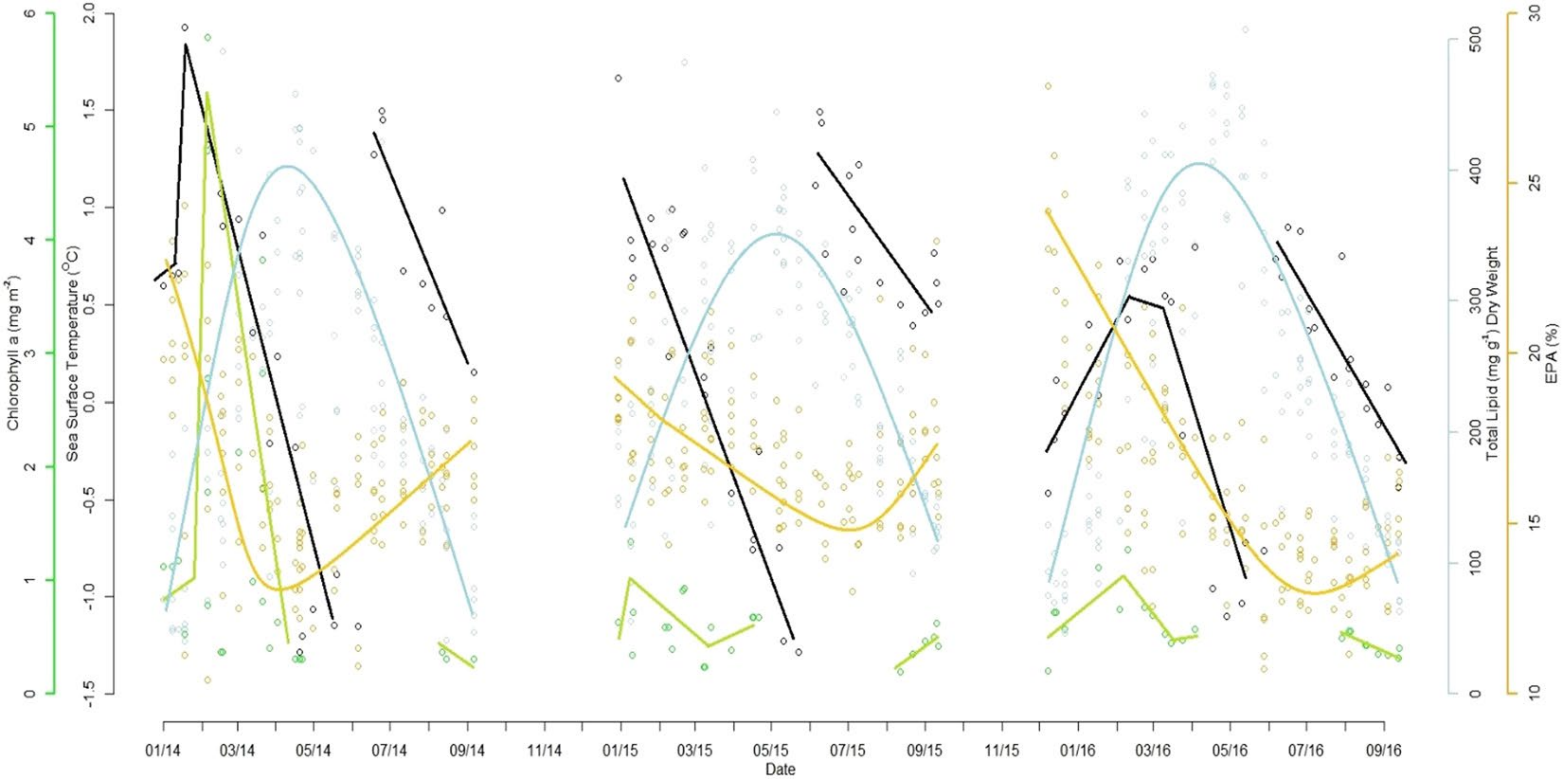
Multi Y axis plot of sea surface temperature (°C; black), total lipid dry weight (mg g^−1^; blue), chlorophyll *a* levels (mg m^−2^; green) and eicosapentaenoic acid (20:5n-3; EPA) percentage (%; yellow) for dates of krill (*Euphausia superba*) sample collection. Lines drawn for illustrative purposes to show general trends.

The inter-relationships between environmental factors showed that TLDW increased as SST decreased in summer and autumn, but TLDW decreased as did SST in winter and spring. This was independent of Chl *a* levels, which were highest in the SOI in 2014, but the highest TLDW levels were in the WAP in 2016 (Fig. 4). The percentage of EPA increased after a decrease in Chl *a* levels (grazing effect), but generally followed the same yearly trends as Chl *a*. EPA was high then decreased in 2014, plateaued in 2015 and again started high and decreased throughout the summer and autumn of 2016 (Fig. 4).

In terms of the fit of models, no models had an adjusted r^2^ value greater than 0.5 for the pooled data of the entire South Atlantic region (Table 1). The CCAMLR region data had multiple models with good fit, with many models exceeding an adjusted r^2^ of 0.5. DHA percentage and 16:1n-7c mass fitted well with the interaction between SST and Chl *a* (8D 3 × 3) (r^2^ = 0.611 and 0.551, respectively) in SG (Supp. Table 1). No fatty acids had an r^2^ above 0.5 at the SOI (Supp. Table 2). DHA percentage was close to fitting with Chl *a* (8D 3 × 3), with an r^2^ value of 0.498 at the WAP (Supp. Table 3). SST and Chl *a* (CCAMLR) fitted to PL and TAG percentages in the WAP (r^2^: 0.564 and 0.559, respectively). EPA percentage fitted well with an adjusted r^2^ of 0.559 for its WAP model of SST and Chl *a* (CCAMLR).

Better fits were found, however, by using the SST and Chl *a* (8D 3 × 3) interaction in the model at the WAP (Supp. Table 3). TLDW, PL and TAG percentages fitted this interaction with adjusted r^2^ values of 0.610, 0.655 and 0.568, respectively. EPA percentage (r^2^: 0.689), and EPA, DHA and 16:1n7c masses (r^2^: 0.615, 0.892, 0.621) all fitted the SST and Chl *a* (8D 3 × 3) interaction at the WAP (Supp Table 3). Additionally, SDA mass correlated with SST and Chl *a* (8D 3 × 3) at the WAP (r^2^: 0.560; Supp. Table 3).

The best model fit of all of those tested was DHA mass at the WAP using the SST and Chl *a* (8D 3 × 3) interaction with an adjusted r^2^ value of 0.892. This can be seen in Supplementary Fig. 3 which compares the slopes and fit of models from the different CCAMLR sub-areas to DHA mass against SST and Chl *a* (CCAMLR) and the 95% confidence interval around the model.

χ^2^ (chi-squared) values are shown for all models, whether they are for the pooled South Atlantic models (Table 1) or for the CCAMLR specific sub-areas (Supp. Tables 1–3).

## Discussion

Krill lipid content and composition, specifically their fatty acid dietary biomarkers, correlate with changes in broad scale environmental data (SST and Chl *a* levels) derived from satellites. Krill eat, metabolise, and store lipids and fatty acids derived from their prey throughout the summer and early autumn when waters are warmer (higher SST) with more available food (diatoms and flagellates; higher Chl *a*)^7,40,48^. In turn, they use these fatty acid and lipid stores during winter and early spring (lower SST and lower Chl *a*), resulting in a decrease in lipid, fatty acid and therefore n-3 LC-PUFA amounts^7,48^. This change of fatty acid composition causes an increase in their other fatty acid composition percentages, although the fatty acid masses may not change. During summer and autumn, decreases in specific fatty acid percentages (EPA, DHA, 16:0 and phytanic acid (derived from phytol, a side chain of chlorophyll)), follow the decrease of SST. This is predominantly due to the overall increase in krill TLDW shifting the fatty acid composition in these seasons to lipids used more for reproduction and over winter survival^8,9,25,50,51^. Similarly, masses of EPA, DHA, 16:0, 16:4n-1, 16:1n-7c and phytanic acid increased through summer and autumn as krill laid down lipid stores for eggs, mostly n-3 LC-PUFA, and increased their TAG percentages prior to winter. This increase in fatty acid mass had an inverse relationship to SST in summer and autumn and may be due to a grazing effect and/or the lag effect of lipids being metabolised, including being stored by krill, after the spring/summer algal bloom^38,52^.

The large increase in Chl *a* seen during the summer of 2014 at SOI was strongly correlated to DHA mass and percentage for that season and year. This suggests that krill were predominantly eating flagellates in the summer of 2014 near the SOI and that this flagellate bloom was detected as extremely elevated green ocean colour and hence elevated Chl *a* levels at the time. Being able to detect the bloom on the same day and at the same location of krill harvest was purely coincidental. Environmental conditions such as sun angle, cloud cover and sea ice did not interfere with ocean colour data capture for that location over that period of time, allowing for one of the best coincidental match ups of environmental data and krill fatty acids throughout the sampling period.

The decrease of SST at SG during winter and spring, however, had an inversely proportional relationship with the increase of these same fatty acid percentages (EPA, DHA, 16:0 and phytanic acid). The fatty acid masses decreased in proportion to the decrease in SST at SG. This may be related to krill using their lipid stores over winter and spring, a time when SST is lower and Chl *a* in the open ocean is lower^18,53^, and most algae is bound in sea ice^18,51,54,55^. However, sea ice is not as prevalent at SG during winter as it is at the WAP and SOI, so herbivorous fatty acid sources would be lacking. The 18:1n-9c/18:1n-7c ratio has been previously shown to move from a more herbivorous diet in the summer/autumn to a more omnivorous diet in winter and spring^7,18,56^. This dietary shift would also be seen as a decrease of fatty acid masses from herbivorous sources (e.g. diatoms and flagellates), however, n-3 LC-PUFA are preferentially conserved in krill as they serve as a major fatty acid functional group for krill health and growth^9,24,57,58^. Therefore, decreases in 16:0, 16:4n-1, 16:1n-7c and phytanic acid masses would be much larger than decreases in EPA and DHA masses, causing their total fatty acid composition percentages to increase proportionally and inversely to SST at this time of year.

Chl *a* levels, indicated from extremely green ocean colour data, could be derived from diatom blooms^30–32^. These blooms may also be from flagellates which are seen as high green ocean colour values too^30,32^, such as those seen at the SOI in the summer of 2014. Chl *a* (8D 3 × 3) levels correlated positively with EPA percentage, and Chl *a* (overall) levels positively related to phytanic acid percentage and mass. However, no flagellate lipid biomarkers in krill were significantly related to Chl *a* data overall, so it is more likely to come from diatom sources, which would then be seen in diatom markers such as EPA, phytanic acid and a high 16:1/16:0 ratio. Diatoms are likely the major source of phytol-derived phytanic acid in this case, as EPA is a dominant FA in diatoms and high 16:1/16:0 and EPA:DHA ratios were observed, reflecting greater diatom abundance^7,18,56,59^. Other diatom markers weren’t as high, possibly due to the differing rates of fatty acid metabolism^60–62^. Biomarkers with faster uptake rates, seen in larger quantities both in the krill's diet and in lipid storage, would be faster to track and could fluctuate more closely to what is seen in the local environment (e.g. small scale blooms and an increase in Chl *a*). EPA and phytanic acid are both readily absorbed and metabolised by krill and can therefore track Chl *a* levels in the environment more closely immediately after a bloom event. EPA can be readily absorbed and stored in the PL of krill. However, some EPA is present (at low levels) within the TAG of krill as well and this may be metabolised even faster than the EPA stored in PL, as it does not need to be converted to PL from the primary dietary source^63^.

Fatty acid biomarker percentages associate better with the Chl *a* (overall) data, however, fatty acid masses are more highly associated with the more specific and localised Chl *a* (8D 3 × 3) data. Krill maintain their percentages of fatty acids between years, seasons and locations^7^, so large scale Chl *a* data might not show smaller fluctuations. Krill fatty acid masses can change dramatically (up to a 10 fold increase between summer and early winter^7^), and hence Chl *a* (8D 3 × 3) data fluctuations are more apparent in localised areas, but not in the overall Chl *a* data. The interaction between SST and Chl *a* (overall) showed a significant relationship to EPA, SDA and 16:0 percentages, while EPA, DHA, and 16:1n-7c masses were more significantly related to the SST and Chl *a* (8D 3 × 3) interaction. Therefore, using different scales of Chl *a* in the SST and Chl *a* interaction within the models can provide a better prediction for either krill fatty acid percentages or masses, depending on the scale of Chl *a* pixel used. Reasons for inconsistencies may be due to differences between regions as these vary with the local environment in that area. These environmental differences will influence the primary production and hence diet of krill in these regions. Therefore, the biomarkers will vary between regions naturally, but may still correlate with the environmental data from that region (e.g. SST and Chl *a* from WAP will correlate with biomarkers from krill within the WAP but not from SOI). Many of the major essential krill fatty acid biomarkers were correlated to SST, Chl *a* and their interaction terms at varying scales.

Tukey tests revealed the interaction with TLDW was mainly driven by SST, and not Chl *a*, and that SST had a close relationship to TLDW in all locations. Because this relationship holds without the Chl *a* interaction term, it can be assumed that SST drives TLDW levels more than Chl *a* levels do. TLDW relates well to SST and Chl *a* (8D 3 × 3) interactions as it is scaled to the krill's weight, which may be affected by both temperature^64^ and its stomach and digestive gland weighing more from being full from chlorophyll rich items^15,57,59^. As TLDW naturally increases throughout summer and autumn and decreases throughout winter and spring^48^, this would also coincidentally inversely follow the decrease of SST in summer and autumn, and decrease proportionally to SST in winter and spring. Therefore, the relationship between SST and TLDW may be coincidental due to the seasonal shift in how krill use their lipids aligning with seasonal shifts in SST. At the regional scale, TLDW was strongly related to Chl *a* (CCAMLR) levels in the SOI and WAP, although not in SG. This may be due to krill having a more herbivorous diet during summer and autumn when krill are harvested from the SOI and WAP and a more omnivorous diet during winter and spring whilst they are harvested at SG.

The interaction between SST and Chl *a* (CCAMLR) was significant for SG, however, and also the WAP, but not for the SOI. This may be due to larger SST shifts at the more extreme ends of the latitudinal scale at WAP and SG^2,65^. These locations would have the greatest extremes in environment, particularly for SST^59,65^, and as such any variations may explain why these locations show a strong relationship between TLDW and the environment. Similarly, at SG, Chl *a* (CCAMLR) was consistently low for all of winter and spring (when able to be recorded), whereas TLDW decreased dramatically during this period, so this may be giving false model fits for this area at this time of year. If readings of Chl *a* levels were possible throughout the winter season, a more closely related trend might be seen.

As TAG is a storage lipid in krill^8,48^, it decreases from late summer through to the following late spring, when krill build stores for both reproduction^50,66^ and survival over winter^8,9,51,53^. This peak in TAG also follows the peak in summer algal blooms (and thus peak Chl *a*) and is seen as a lag effect. This lag is due to both the time it takes for krill to metabolise TAG and the rate that krill convert fatty acids in algal TAG to their own PL stores. The PL percentages in krill tracked with Chl *a* (overall and 8D 3 × 3). PL has also been reported as a storage lipid in krill^8^, and is known to be vital for the storage of essential n-3 LC-PUFA for reproduction^25,52,66,67^ and krill health^16,68^. Interestingly, PL levels were not related to changes in SST, but tracked well with changes in Chl *a*, possibly due to the way that polar phytoplankton blooms can occur via a boom-bust cycle^38^. Many types of algae in polar latitudes are high in PL^17,21,69,70^, and are also very green in colour, which could be one reason why krill PL are so closely related to Chl *a* levels. PL can be incorporated very quickly into krill tissue as krill predominantly store their lipids as PL^8^. Therefore, there would be little lag between ingestion, metabolism and incorporation. Our data shows there is little to no lag seen between Chl *a* data and PL percentages.

The EPA:DHA ratio in krill was significantly related to Chl *a* (8D 3 × 3). A higher EPA:DHA ratio suggests that krill are consuming more diatoms than flagellates in their diet^7,19^. This relationship could be due to diatom and flagellate blooms affecting the ocean colour readings (higher green values) from satellite more so than other factors^30,31,71^. The green colour can be detected relatively easily in the ocean^72^ and ocean colour is changing far faster with the climate than was predicted by models^34^. These changes in plankton community assemblages could also be changing faster than expected^33,35^, which would be reflected in a changing EPA:DHA ratio. Krill potentially prefer a more diatom-based diet when blooms occur, even if flagellates are available in the water column^33,38,53^. Such a dietary preference might skew the EPA:DHA ratio of krill, and could be related to the amount of Chl *a* being detected via remote sensing^38^, whether at the 8D 3 × 3 or overall scale. The 16:1/16:0 ratio shows differences in plankton types being consumed by krill^7,56^, and was not associated with any Chl *a* or SST data over the whole South Atlantic, but did show differences at smaller CCAMLR region scales. This may be due to krill diet shifting with seasons at the same time as the krill fishery also shifts it's fishing location at the end of autumn (from SOI or WAP) to the start of winter (SG).

The krill fishery operates at SG during winter and spring when algal populations are naturally lower in the water column^18,53^, and most algae is bound in sea ice^18,51,54,55^. Remote sensing during winter in polar regions is particularly hard for multiple reasons e.g. cloud cover, sea ice, and sun angle^72^. These difficulties create gaps in the Chl *a* data available at SG, which may be falsely lowering these levels. Ground truthing the Chl *a* concentrations in SG throughout the winter season is one way of confirming this remote sampling data bias. Large-scale and long-term studies such as the Palmer Long-Term Ecosystem Research (LTER) program^41,42,73,74^ and the U.S. Antarctic Marine Living Resources (AMLR) program^42,75^ can ground truth their Chl *a* recordings by being present year round in a location to take water samples for Chl *a* analysis. Future technological advancements may also assist with such ground-truthing, including the use of deployed moorings that take water samples and record algal fluorescence year-round. Improving satellite algorithms for ocean colour data to be converted into Chl *a* concentrations in polar regions would significantly increase the number of data points available throughout winter. In turn, such enhanced data could then be better related to other factors, such as krill diet, with the remote sensed Chl *a* data being closer to the true Chl *a* levels present at that time of year.

Additionally, due to the occurrence of krill swarming, the spatial and temporal scales used may not be the tightest to fit krill dynamics as more than a single krill aggregation may be present in a 3 km × 3 km grid, and krill diet may vary greatly over an 8 Day period. However, total lipid content varies slowly, particularly in the whole animal, as does their fatty acid profile. So, large dietary differences in krill aren’t expected to be seen at this level over the period of 8 days or at a scale of 3 km × 3 km. If, however, this analysis was using the lipids and fatty acids of krill stomachs or digestive glands, then this may have a more significant impact as these would change greatly over the period of 8 days and would show variation within a 3 km × 3 km grid.

This study used satellite-derived ocean colour and SST data in conjunction with fatty acid content and composition in krill diet at different times of year and in different locations. Cross-disciplinary work such as in this study is promising as it enables remote sensing and satellite oceanography specialists to better link with biological, physiological and ecological specialists. This collaboration may enable issues such as winter sampling of Chl *a* through satellites to be better understood, and solutions to issues such as sea ice and cloud cover to be resolved at a scale that is meaningful to the biology associated to that Chl *a* reading, whether primary producers or krill.

The relationship between SST, Chl *a* and krill lipid biochemistry presented here could be expanded to examine similar relationships in krill diet and krill lipid content and composition in other regions around Antarctica. The approach could also be used for other marine-based species both in the Antarctic and other polar areas where sampling is restricted.

## Methods

### Krill sample collection and analysis

Krill lipid and fatty acid data used for this analysis are published in Hellessey, *et al*.^48^ and Ericson, *et al*.^7^. Krill samples for which lipid and fatty acid data were derived were collected and analysed fortnightly from 3 adult male and 3 adult female krill, with samples collected by the fishing industry (FV *Saga seas*) in the South Atlantic Ocean (Area 48) (N = 391). Samples from January – May 2014 to 2016 from the WAP (Area 48.1) and SOI (Area 48.2), and June – September 2014 to 2016 from SG (Area 48.3) were used for this analysis. Boundaries and area size for the CCAMLR fishing sub-areas can be found at www.ccamlr.org. The mean body length (Standard length 1^76^) of krill was 46.0 mm (±4.8) and the mean dry mass was 0.16 g (±0.05). The total lipid (mg g^−1^ dry weight) of krill (TLDW) in each season of each year is given in Table 2.

**Table 2:** Average total lipid (mg g^−1^ dry weight, mean ± SD), length (mm) and weight (g) of *Euphausia superba* by sex, season and year.

	Total lipid content (mg g^−1^ dry weight)		Average length (mm)	Average dry weight (g)
	Males (N = 190)	Females (N = 201)		
Summer 2014	148.9 ± 116.2	148.0 ± 58.7	48.19	0.21
Autumn 2014	329.8 ± 68.3	268.6 ± 101.3	47.23	0.17
Winter 2014	210.6 ± 86.2	203.8 ± 66.4	42.04	0.11
Spring 2014	59.8 ± 12.7	114.8 ± 30.0	45.66	0.14
Summer 2015	168.1 ± 130.1	166.7 ± 102.1	46.49	0.17
Autumn 2015	309.2 ± 60.8	303.8 ± 74.2	45.63	0.14
Winter 2015	234.1 ± 60.7	233.5 ± 64.3	48.42	0.17
Spring 2015	138.9 ± 17.2	131.3 ± 16.5	48.95	0.20
Summer 2016	271.8 ± 128.3	217.2 ± 94.3	46.77	0.19
Autumn 2016	399.7 ± 51.8	361.3 ± 96.6	48.51	0.21
Winter 2016	209.0 ± 46.1	208.0 ± 57.2	44.01	0.13
Spring 2016	90.7 ± 26.6	120.1 ± 9.3	42.97	0.13

Seasons are defined as summer (1 December to 28 February), autumn (1 March to 31 May), winter (1 June to 31 August), and spring (1 September to 30 November).

### Satellite data extraction and analysis

This study used ocean colour data from the NASA Moderate Resolution Imaging Spectroradiometer Aqua (MODIS-Aqua) L3 mapped data products (https://oceancolor.gsfc.nasa.gov/data/aqua/) and sea surface temperature data from the GHRSST L4 gridded products (https://data.nodc.noaa.gov/ghrsst/L4/). The sea surface temperature data and the 3 different ocean colour Remote Sensed Reflectance wavelengths (RRS; red, green and blue: 443, 488 and 555 nm, respectively) were extracted for each date krill were collected in an area in the South Atlantic Ocean (bounds of 55–80°S and 30–80°W) in a 1 km × 1 km grid of pixels. The exact GPS location of krill collection on that date was then used to extract the pixel value (28 successful matches = 4.18% matched), but due to the low match-up rate this was expanded both temporally and spatially to an 8-day average and a 3 × 3 pixel area (145 matches = 21.64% matched, Table 3). Whenever data was patchy it was smoothed linearly to the nearest pixel within a 4 km area.

**Table 3:** Decision table used for temporal and spatial fields for the red, green and blue wavelengths in Moderate Resolution Imaging Spectroradiometer (MODIS) to generate chlorophyll *a* data.

Case	Temporal Averaging	Pixel averaging	Percentage (%) match
1	Daily	1 km × 1 km	4.18 (28)
2	Daily	3 km × 3 km	7.01 (47)
3	8 Day	1 km × 1 km	21.64 (145)
4	8 Day	3 km × 3 km	27.91 (187)
5	8 Day	Custom	WAP – 46.26 (310)
		CCAMLR	SOI – 51.34 (344)
		Regions	SG – 66.86 (448)

The Commission for Conservation of Antarctic Marine Living Resources (CCAMLR) regions were defined as the West Antarctic Peninsula (WAP, Area 48.1), the South Orkney Islands (SOI, Area 48.2) and South Georgia (SG, Area 48.3) (www.ccamlr.org). Raw value for percent data match in brackets. Total number of days with lipid data to match against = 670.

The ocean colour RRS data were converted into Chl *a* concentrations using the MODIS Southern Ocean chlorophyll algorithm in Johnson, *et al*.^31^. Once converted into Chl *a* concentrations, this environmental data (SST and Chl *a* values) was merged into the same data frame as the lipid data by matching the date and GPS location of 1 day 1 × 1 pixel locations to the date and GPS location of krill harvest. To examine the seasonal trend for Chl *a*, each CCAMLR fishing sub-area within the South Atlantic (Area 48) also had its Chl *a* calculated for 8-day averages. These wider geographic areas of the WAP, the SOI and SG generated much higher recovery rates for Chl *a* (46.26%, 51.34% and 66.86% respectively, Table 3) and is hereon called Chl *a* (CCAMLR). This data was also merged into the same data frame by matching dates and GPS locations of krill sampling, as done previously. However, data for some dates did not match krill harvesting location (e.g. Chl *a* data from SG on a day when krill were collected from WAP), so the rates of Chl *a* recovery decreased once matched to krill lipid data. Therefore, to achieve the best Chl *a* matches to lipid data, the Chl *a* concentration was kept in a hierarchy from Case 1 to 5 (Table 3). For example, if a daily pixel match was available, this was kept in preference over an 8-day 3 × 3 or 8-day regional Chl *a* concentration. This method increased the overall match rate to 226 matches out of the 373 lipid data point = 60.59% matched. This merged Chl *a* data (hereon called Chl *a* (overall)) was used to examine the larger scale trends in Chl *a* across the entire south Atlantic Ocean and krill lipids.

### Data and statistical analysis

Statistical analysis was done in RStudio (version 1.0.153^©^ 2017, packages nlme, ggplot2, ggmap, anytime, and reshape2). Multifactorial ANOVAs were performed using SST and Chl *a*, as well as their interaction terms, as factors for the variables of total lipid content (mg g^−1^ dry weight), PL and TAG percentage, individual fatty acid percentage and mass data for the fatty acids most associated with primary production (mostly diatom and flagellate markers), and the ratios of 16:1/16:0 and EPA:DHA. These were performed to examine diatom levels (higher EPA, higher 16:1)^7^ and to look at diatom or flagellate dominance in the diet (higher DHA, lower 16:1)^7^, for each location and season. Data were log or square root transformed when the assumptions of normality and homogeneity of variances were not met - Chl *a* is typically log distributed. Linear models were similarly produced using the same factors as for the multifactorial ANOVAs, including interaction terms, but sub-divided into summer/autumn and winter/spring models due to the large seasonal shift in SST arising from the change of harvesting location by the FV *Saga Seas*. Models were tested for fit using a standard regression table, where the adjusted r^2^ value showed the fit of points to the confidence interval of the model. Models were additionally run through drop testing and Tukey post-hoc tests to ensure no compounding of results was occurring. Models of best fit had adjusted r^2^ values >0.5, a P value of <0.05 from the multifactorial ANOVAs and a χ^2^ value above 0.1. These models are shown within all tables throughout the Results and Supplementary Materials as greyed out.

Maps were produced within R using the maps and ggmap packages to see the geographic distribution of krill lipid content (mg g^−1^ dry weight) as well as SST and Chl *a*.

## Supplementary information

Supplementary Materials.
