# Supplementary material for: Antarctic Krill Lipid and Fatty acid Content Variability is Associated to Satellite Derived Chlorophyll a and Sea Surface Temperatures
Source: Sci Rep. 2020 Apr 8;10:6060. doi: 10.1038/s41598-020-62800-7 (PMC7142126; doi:10.1038/s41598-020-62800-7)
Supplement: Supplementary file 1 — Supplementary Materials. [file 41598_2020_62800_MOESM1_ESM.docx]

**Antarctic Krill Lipid and Fatty acid Content Variability is associated to Satellite Derived Chlorophyll *a* and Sea Surface Temperatures**

Nicole Hellessey, Robert Johnson, Jessica A. Ericson, Peter D. Nichols, So Kawaguchi, Stephen Nicol, Nils Hoem and Patti Virtue

**Supplementary Table 1:** Euphausia superba (collected from South Georgia) total lipid (mg g^-1^) dry weight (TLDW), lipid class (phospholipid (PL) and triacylglycerol (TAG)) and fatty acid (20:5n-3 (EPA), 22:6n-3 (DHA) and 18:4n-3 (SDA)) percentage composition (%) and mass (ug) in relation to sea surface temperature (SST), chlorophyll (Chl a) and their interaction terms. Chl a was measured at both a Commission for the Conservation of Antarctic Marine Living Resources (CCAMLR) region wide scale (CCAMLR region) and at an 8-day 3 km x 3 km (8D 3x3) pixel scale. Values given are for: P values, r^2^ values (italics) and χ^2^ values (bold) for the model of best fit. Cells that are greyed out have a P value < 0.05, an r^2^ of >0.5 and a χ^2^ value > 0.1.

|  | SST | Chl *a (CCAMLR)* | Chl *a* (8D 3x3) | SST*Chl *a (CCAMLR)* | SST*Chl *a* (8D 3x3) |
| --- | --- | --- | --- | --- | --- |
| TLDW (mg g^-1^) | 0.003 (*0.054*) **0.246** | 0.219 (*0.008*) **0.240** | 0.084 (*0.095*) **0.236** | < 0.001 (*0.299*) **0.240** | 0.336 (*0.337*) **0.236** |
| PL % | 0.103 (*0.115*) **0.264** | 0.013 (*0.083*) **0.249** | 0.019 (*0.195*) **0.236** | 0.001 (*0.297*) **0.249** | 0.001 (*0.301*) **0.236** |
| TAG % | 0.206 (*0.004*) **0.102** | 0.010 (*0.088*) **0.249** | 0.083 (*0.095*) **0.241** | 0.001 (*0.292*) **0.249** | 0.163 (*0.194*) **0.241** |
| EPA % | 0.095 (*0.012*) **0.241** | 0.083 (*0.033*) **0.078** | 0.001 (*0.419*) **0.236** | 0.002 (*0.235*) **0.240** | 0.003 (*0.437*) **0.236** |
| EPA (ug) | 0.035 (*0.023*) **0.239** | 0.105 (*0.027*) **0.239** | 0.178 (*0.041*) **0.236** | 0.001 (*0.179*) **0.239** | 0.645 (*0.032*) **0.236** |
| DHA % | 0.863 (-*0.007*) **0.241** | 0.003 (*0.120*) **0.240** | 0.001 (*0.377*) **0.236** | 0.001 (*0.280*) **0.240** | <0.001 (*0.611*) **0.236** |
| DHA (ug) | 0.026 (*0.029*) **0.239** | 0.602 (-*0.012*) **0.239** | 0.052 (*0.128*) **0.236** | 0.001 (*0.149*) **0.239** | 0.817 (*0.072*) **0.236** |
| SDA % | 0.005 (*0.046*) **0.087** | 0.076 (*0.035*) **0.019** | 0.594 (-*0.033*) **0.236** | 0.583 (*0.121*) **0.085** | 0.991 (*0.062*) **0.236** |
| SDA (ug) | 0.007 (*0.042*) **0.239** | 0.083 (-*0.010*) **0.239** | 0.551 (*0.095*) **0.236** | 0.409 (*0.162*) **0.238** | 0.029 (*0.269*) **0.236** |
| 16:0 % | 0.173 (*0.006*) **0.241** | 0. 018 (*0.072*) **0.242** | 0.036 (*0.155*) **0.246** | 0.022 (*0.163*) **0.242** | 0.642 (*0.216*) **0.246** |
| 16:0 (ug) | 0.277 (*0.001*) **0.239** | 0.008 (*0.095*) **0.239** | 0.001 (*0.381*) **0.236** | 0.001 (*0.281*) **0.239** | 0.001 (*0.497*) **0.236** |
| 16:4n-1 % | < 0.001 (*0.104*) **0.004** | 0.236 (*0.007*) **0.004** | 0.046 (*0.137*) **0.023** | 0.903 (*0.136*) **0.004** | 0.314 (*0.122*) **0.023** |
| 16:4n-1 (ug) | 0.001 (*0.077*) **0.239** | 0.841 (-*0.016*) **0.239** | 0.481 (-*0.022*) **0.236** | 0.030 (*0.119*) **0.239** | 0.472 (-*0.050*) **0.236** |
| 16:1n-7c % | 0.756 (-*0.006*) **0.241** | 0.001 (*0.103*) **0.239** | 0.007 (*0.265*) **0.236** | 0.001 (*0.279*) **0.239** | 0.956 (*0.247*) **0.236** |
| 16:1n-7c (ug) | 0.330 (-*0.000*) **0.239** | 0.003 (*0.125*) **0.239** | 0.001 (*0.443*) **0.236** | < 0.001 (*0.336*) **0.239** | <0.001 (*0.551*) **0.236** |
| 16:1/16:0 ratio (ug) | 0.985 (-*0.007*) **0.239** | 0.015 (*0.078*) **0.239** | 0.020 (*0.195*) **0.236** | 0.004 (*0.225*) **0.239** | 0.978 (*0.127*) **0.236** |
| EPA/DHA ratio (ug) | 0.245 (*0.002*) **0.239** | 0.004 (*0.114*) **0.239** | 0.949 (-*0.047*) **0.236** | 0.033 (*0.155*) **0.239** | 0.398 (*0.029*) **0.236** |
| Phytol % | 0.252 (*0.002*) **<0.001** | 0.306 (*0.001*) **<0.001** | 0.859 (-*0.046*) **<0.001** | 0.507 (*0.015*) **<0.001** | 0.691 (-*0-070*) **<0.001** |
| Phytol (ug) | 0.059 (*0.018*) **<0.001** | 0.783 (-*0.015*) **<0.001** | 0.482 (-*0.023*) **<0.001** | 0.603 (-*0.008*) **<0.001** | 0.387 (-*0.013*) **<0.001** |

**Supplementary Table 2:** Euphausia superba (collected from South Orkney Islands) total lipid (mg g^-1^) dry weight (TLDW), lipid class (phospholipid (PL) and triacylglycerol (TAG)) and fatty acid (20:5n-3 (EPA), 22:6n-3 (DHA) and 18:4n-3 (SDA)) percentage composition (%) and mass (ug) in relation to sea surface temperature (SST), chlorophyll a (Chl a) and their interaction terms. Chl a was measured at both a Commission for the Conservation for Antarctic Marine Living Resources (CCAMLR) region wide scale (CCAMLR region) and at an 8-day 3 km x 3 km (8D 3x3) pixel scale. Values given are for: P values, r^2^ values (italics) and χ^2^ values (bold) for the model of best fit. Cells that are greyed out have a P value < 0.05, an r^2^ of >0.5 and a χ^2^ value > 0.1.

|  | SST | Chl *a (CCAMLR)* | Chl *a (8D 3x3)* | SST*Chl *a (CCAMLR)* | SST*Chl *a (8D 3x3)* |
| --- | --- | --- | --- | --- | --- |
| TLDW (mg g^-1^) | < 0.001 (*0.168*) **0.081** | 0.003 (*0.059*) **0.018** | 0.124 (*0.034*) **0.238** | < 0.001 (*0.217*) **0.081** | 0.672 (*0.084*) **0.238** |
| PL % | 0.652 (-*0.006*) **0.028** | 0.001 (*0.102*) **0.005** | 0.001 (*0.245*) **0.251** | 0.098 (*0.118*) **0.027** | 0.006 (*0.209*) **0.251** |
| TAG % | 0.004 (*0.054*) **0.006** | < 0.001 (*0.121*) **<0.001** | 0.731 (*-0.021*) **0.085** | 0.049 (*0.164*) **0.007** | 0.587 (-*0.041*) **0.085** |
| EPA % | 0.001 (*0.082*) **0.079** | 0.001 (*0.078*) **0.017** | 0.002 (*0.188*) **0.241** | 0.296 (*0.112*) **0.080** | 0.021 (*0.157*) **0.241** |
| EPA (ug) | < 0.001 (*0.122*) **0.239** | 0.229 (*0.004*) **0.239** | 0.007 (*0.146*) **0.238** | 0.082 (*0.184*) **0.239** | 0.029 (*0.142*) **0.238** |
| DHA % | 0.001 (*0.092*) **0.081** | 0.016 (*0.037*) **0.018** | 0.198 (*0.017*) **0.241** | 0.426 (*0.102*) **0.081** | 0.364 (-*0.010*) **0.241** |
| DHA (ug) | < 0.001 (*0.123*) **0.239** | 0.070 (*0.018*) **0.239** | 0.529 (-*0.014*) **0.238** | 0.345 (*0.186*) **0.239** | 0.594 (*0.026*) **0.238** |
| SDA % | 0.121 (*0.010*) **0.081** | 0.152 (*0.008*) **0.018** | 0.122 (*0.034*) **0.241** | 0.263 (*0.065*) **0.081** | 0.042 (*0.126*) **0.241** |
| SDA (ug) | 0.001 (*0.089*) **0.239** | 0.057 (-*0.003*) **0.239** | 0.417 (*0.057*) **0.238** | 0.910 (*0.139*) **0.239** | 0.415 (*0.034*) **0.238** |
| 16:0 % | 0.007 (*0.045*) **0.079** | 0.063 (*0.019*) **0.017** | 0.292 (*0.003*) **0.241** | 0.997 (*0.032*) **0.080** | 0.501 (-*0.035*) **0.241** |
| 16:0 (ug) | < 0.001 (*0.143*) **0.239** | 0.095 (*0.014*) **0.239** | 0.382 (-*0.005*) **0.238** | 0.011 (*0.209*) **0.239** | 0.635 (-*0.017*) **0.238** |
| 16:4n-1 % | 0.305 (*0.000*) **0.082** | 0.819 (-*0.007*) **0.019** | 0.045 (*0.073*) **0.241** | 0.606 (-*0.021*) **0.083** | < 0.001 (*0.367*) **0.241** |
| 16:4n-1 (ug) | 0.051 (*0.021*) **0.239** | 0.541 (-*0.005*) **0.239** | 0.003 (*0.171*) **0.238** | 0.263 (*0.052*) **0.239** | 0.001 (*0.280*) **0.238** |
| 16:1n-7c % | 0.111 (*0.011*) **0.079** | 0.027 (*0.030*) **0.017** | 0.496 (-*0.013*) **0.241** | 0.415 (*0.024*) **0.080** | 0.078 (*0.028*) **0.241** |
| 16:1n-7c (ug) | < 0.001 (*0.115*) **0.239** | 0.090 (*0.015*) **0.239** | 0.573 (-*0.016*) **0.238** | 0.002 (*0.192*) **0.239** | 0.266 (-*0.016*) **0.238** |
| 16:1/16:0 ratio (ug) | 0.289 (*0.001*) **0.239** | 0.072 (*0.017*) **0.239** | 0.751 (-*0.022*) **0.238** | 0.320 (*0.010*) **0.239** | 0.077 (*0.024*) **0.238** |
| EPA/DHA ratio (ug) | 0.686 (-*0.006*) **0.239** | 0.482 (-*0.004*) **0.239** | 0.001 (*0.271*) **0.238** | 0.522 (-*0.016*) **0.239** | 0.001 (*0.285*) **0.238** |
| Phytol % | 0.046 (*0.022*) **<0.001** | 0.353 (-*0.001*) **<0.001** | 0.507 (-*0.013*) **0.001** | 0.549 (*0.019*) **<0.001** | 0.457 (-*0.001*) **0.001** |
| Phytol (ug) | 0.199 (*0.005*) **<0.001** | 0.246 (*0.003*) **<0.001** | 0.507 (-*0.013*) **0.001** | 0.706 (*0.002*) **<0.001** | 0.457 (-*0.001*) **0.001** |

**Supplementary Table 3:** Euphausia superba (collected from the West Antarctic Peninsula) total lipid (mg g^-1^) dry weight (TLDW), lipid class (phospholipid (PL) and triacylglycerol (TAG)) and fatty acid (20:5n-3 (EPA), 22:6n-3 (DHA) and 18:4n-3 (SDA)) percentage composition (%) and mass (ug) in relation to sea surface temperature (SST), chlorophyll a (Chl a) and their interaction terms. Chl a was measured at both a Commission for the Conservation of Antarctic Marine Living Resources (CCAMLR) region wide scale (CCAMLR region) and at an 8-day 3 km x 3 km (8D 3x3) pixel scale. Values given are for: P values, r^2^ values (italics) and χ^2^ values (bold) for the model of best fit. Cells that are greyed out have a P value < 0.05, an r^2^ of >0.5 and a χ^2^ value > 0.1.

|  | SST | Chl *a (CCAMLR)* | Chl *a (8D 3x3)* | SST*Chl *a (CCAMLR)* | SST*Chl *a (8D 3x3)* |
| --- | --- | --- | --- | --- | --- |
| TLDW (mg g^-1^) | < 0.001 (*0.304*) **0.240** | 0.014 (*0.148*) **0.238** | 0.185 (*0.059*) **0.235** | 0.001 (*0.363*) **0.238** | 0.002 (*0.610*) **0.235** |
| PL % | 0.006 (*0.077*) **0.028** | 0.004 (*0.212*) **0.089** | 0.949 (-*0.071*) **0.235** | < 0.001 (*0.564*) **0.089** | 0.001 (*0.655*) **0.235** |
| TAG % | 0.007 (*0.073*) **0.102** | 0.001 (*0.277*) **0.023** | 0.782 (-*0.065*) **0.235** | < 0.001 (*0.559*) **0.089** | 0.004 (*0.568*) **0.235** |
| EPA % | 0.001 (*0.137*) **0.240** | 0.012 (*0.157*) **0.238** | 0.388 (-*0.014*) **0.235** | < 0.001 (*0.559*) **0.238** | < 0.001 (*0.689*) **0.235** |
| EPA (ug) | < 0.001 (*0.217*) **0.239** | 0.079 (*0.065*) **0.238** | 0.059 (*0.175*) **0.235** | 0.001 (*0.311*) **0.238** | 0.002 (*0.615*) **0.235** |
| DHA % | 0.001 (*0.122*) **0.239** | 0.254 (*0.010*) **0.238** | 0.001 (*0.498*) **0.235** | 0.028 (*0.148*) **0.238** | 0.014 (*0.467*) **0.235** |
| DHA (ug) | < 0.001 (*0.285*) **0.239** | 0.357 (-*0.004*) **0.238** | 0.624 (-*0.053*) **0.235** | < 0.001 (*0.430*) **0.238** | < 0.001 (*0.892*) **0.235** |
| SDA % | 0.795 (-*0.011*) **0.083** | 0.187 (*0.024*) **0.086** | 0.009 (*0.351*) **0.235** | 0.541 (*0.098*) **0.087** | 0.068 (*0.295*) **0.235** |
| SDA (ug) | < 0.001 (*0.311*) **0.239** | 0.020 (*0.131*) **0.238** | 0.372 (-*0.010*) **0.235** | 0.001 (*0.390*) **0.238** | 0.004 (*0.560*) **0.235** |
| 16:0 % | 0.001 (*0.122*) **0.240** | 0.048 (*0.089*) **0.238** | 0.012 (*0.325*) **0.235** | 0.014 (*0.214*) **0.238** | 0.014 (*0.464*) **0.235** |
| 16:0 (ug) | < 0.001 (*0.254*) **0.239** | 0.059 (*0.078*) **0.238** | 0.097 (*0.126*) **0.235** | 0.001 (*0.347*) **0.238** | 0.002 (*0.621*) **0.235** |
| 16:4n-1 % | 0.527 (-*0.007*) **0.021** | 0.945 (-*0.031*) **0.245** | 0.004 (*0.412*) **0.235** | 0.982 (*0.189*) **0.245** | 0.003 (*0.589*) **0.235** |
| 16:4n-1 (ug) | < 0.001 (*0.197*) **0.239** | 0.059 (*0.079*) **0.238** | 0.041 (*0.213*) **0.235** | 0.008 (*0.280*) **0.238** | 0.093 (0.253) **0.235** |
| 16:1n-7c % | 0.130 (*0.015*) **0.240** | 0.625 (-*0.023*) **0.240** | 0.003 (*0.446*) **0.235** | 0.104 (*0.043*) **0.241** | 0.026 (*0.406*) **0.235** |
| 16:1n-7c (ug) | < 0.001 (*0.185*) **0.239** | 0.083 (*0.063*) **0.238** | 0.080 (*0.145*) **0.235** | 0.001 (*0.302*) **0.238** | 0.014 (*0.468*) **0.235** |
| 16:1/16:0 ratio (ug) | 0.445 (-*0.005*) **0.239** | 0.897 (-*0.031*) **0.238** | 0.001 (*0.489*) **0.235** | 0.192 (*0.012*) **0.238** | 0.017 (*0.449*) **0.235** |
| EPA/DHA ratio (ug) | 0.031 (*0.043*) **0.239** | 0.181 (*0.026*) **0.238** | 0.003 (*0.436*) **0.235** | 0.188 (*0.162*) **0.238** | 0.040 (*0.358*) **0.235** |
| Phytol % | 0.426 (-*0.004*) **<0.001** | 0.013 (*0.156*) **<0.001** | 0.859 (-*0.074*) **<0.001** | 0.039 (*0.246*) **<0.001** | 0.072 (*0.308*) **<0.001** |
| Phytol (ug) | 0.816 (-*0.011*) **<0.001** | 0.014 (*0.149*) **<0.001** | 0.799 (-*0.066*) **<0.001** | 0.145 (*0.185*) **<0.001** | 0.046 (*0.342*) **<0.001** |


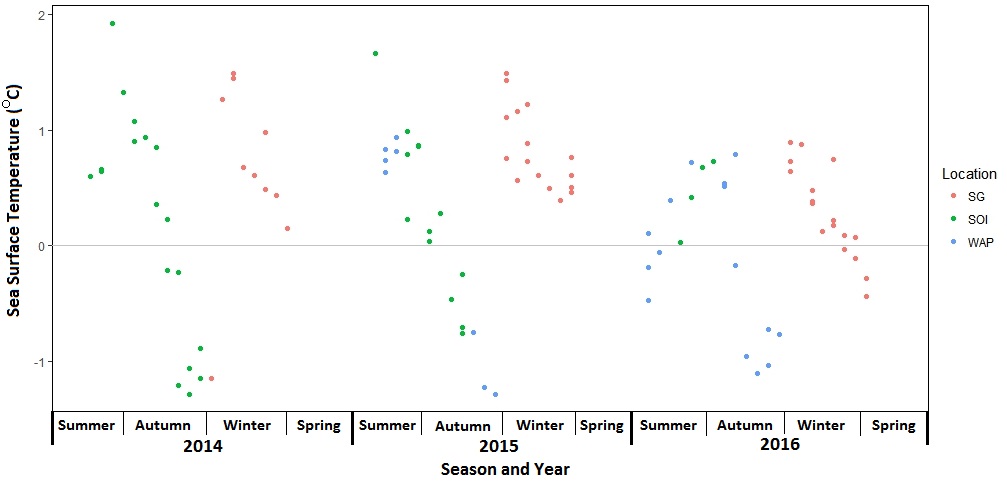


**Supplementary Figure 1:** Sea surface temperatures (°C) from January 2014 – September 2016 coloured by *Euphausia superba* sample location (SG: South Georgia, SOI: South Orkney Islands, WAP: West Antarctic Peninsula). The x-axis is the season and year of krill sample collection. The seasons are defined as: Summer (December 1 – February 28), autumn (March 1 – May 31), winter (June 1 – August 31) and spring (September 1 – November 30).


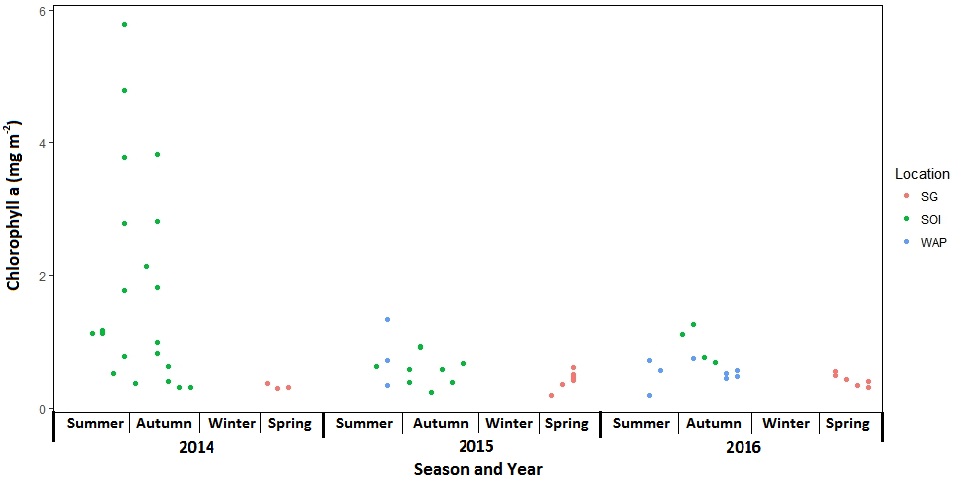


**Supplementary Figure 2:** Chlorophyll *a* concentrations (mg m^-2^) from January 2014 – September 2016 coloured by *Euphausia superba* sample location (SG: South Georgia, SOI: South Orkney Islands, WAP: West Antarctic Peninsula). The x-axis is season and year of krill sample collection. The seasons are defined as: Summer (December 1 – February 28), autumn (March 1 – May 31), winter (June 1 – August 31) and spring (September 1 – November 30).


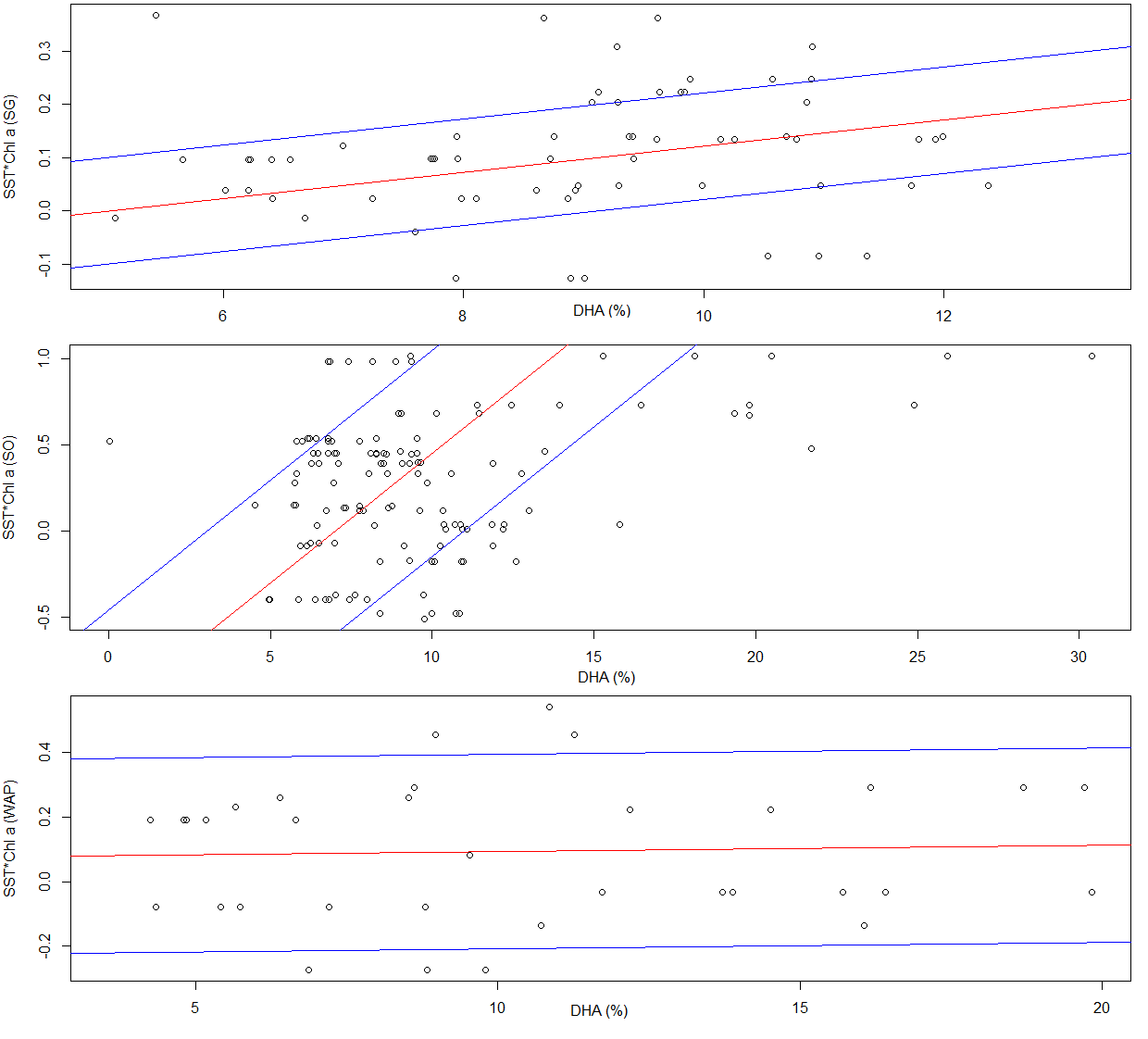


**Supplementary Figure 3:** Slopes of the models of best fit (red) and the 95% confidence interval for that model (blue) for docosahexaenoic acid (DHA; 22:6n-3) percentage (%) in *Euphausia superba* sampled in the different Commission for the Conservation of Antarctic Marine Living Resources (CCAMLR) sub-areas (West Antarctic Peninsula (WAP), South Orkney Islands (SOI) and South Georgia (SG)) against sea surface temperature (°C, SST) and chlorophyll *a* (mg m^2^, Chl *a*).
